# Cultivation of gastrointestinal microbiota in a new growth system revealed dysbiosis and metabolic disruptions in carcinoma-bearing rats

**DOI:** 10.3389/fmicb.2022.949272

**Published:** 2022-09-02

**Authors:** Betsy Anaid Peña-Ocaña, Yuki Hoshiko, Mayel Silva-Flores, Toshinari Maeda, Israel Pérez-Torres, Rodolfo García-Contreras, Wilbert Gutiérrez-Sarmiento, Luz Hernández-Esquivel, Álvaro Marín-Hernández, Rosina Sánchez-Thomas, Emma Saavedra, José Salud Rodríguez-Zavala, Ricardo Jasso-Chávez

**Affiliations:** ^1^Departamento de Bioquímica, Instituto Nacional de Cardiología Ignacio Chávez, Mexico City, Mexico; ^2^Division of Environment-Conscious Chemistry and Bioengineering, Department of Biological Functions Engineering, Kyushu Institute of Technology, Kitakyushu, Japan; ^3^Departamento de Biomedicina Cardiovascular, Instituto Nacional de Cardiología Ignacio Chávez, Mexico City, Mexico; ^4^Departamento de Microbiología y Parasitología, Facultad de Medicina, Universidad Nacional Autónoma de México, Mexico City, Mexico; ^5^Instituto Tecnológico Nacional de México/Instituto Tecnológico de Tuxtla Gutiérrez, Tuxtla Gutiérrez, Chiapas, Mexico

**Keywords:** anaerobic culture, ionomics, methane production, oxidative stress, biofilm, metabarcoding, kinetic analysis

## Abstract

A challenge in the study of gastrointestinal microbiota (GITm) is the validation of the genomic data with metabolic studies of the microbial communities to understand how the microbial networks work during health and sickness. To gain insights into the metabolism of the GITm, feces from healthy and sick rats with cancer were inoculated in a defined synthetic medium directed for anaerobic prokaryote growth (INC-07 medium). Significant differences between cultures of healthy and sick individuals were found: 1) the consumption of the carbon source and the enzyme activity involved in their catabolism (e.g., sucrase, lactase, lipases, aminotransferases, and dehydrogenases); 2) higher excretion of acetic, propionic, isobutyric, butyric, valeric, and isovaleric acids; 3) methane production; 4) ability to form biofilms; and 5) up to 500 amplicon sequencing variants (ASVs) identified showed different diversity and abundance. Moreover, the bowel inflammation induced by cancer triggered oxidative stress, which correlated with deficient antioxidant machinery (e.g., NADPH-producing enzymes) determined in the GITm cultures from sick individuals in comparison with those from control individuals. Altogether, the data suggested that to preserve the microbial network between bacteria and methanogenic archaea, a complete oxidation of the carbon source may be essential for healthy microbiota. The correlation of 16S rRNA gene metabarcoding between cultures and feces, as well as metabolomic data found in cultures, suggest that INC-07 medium may be a useful tool to understand the metabolism of microbiota under gut conditions.

## Introduction

Gastrointestinal microbiota (GITm) studies have gained relevance due to the findings that it can be associated with the integral health; indeed, changes in composition and microbial abundance of the microbiota have been to the development of some diseases (Malard et al., 2021). The microbial communities found in microbiota show symbiotic and mutualistic behaviors not only among them but also with host cells. Dysbiosis is a term that has been applied to an unhealthy condition that involves the imbalance, dysfunction, or alteration of the GITm, and it has been correlated with the development of different types of cancer, metabolic and heart diseases, infections, etc., (Wei et al., 2021). In this regard, there is evidence that some pathologies, such as inflammatory bowel disease or metabolic syndrome, provoke important alterations in the gastrointestinal microbiota composition since they occur with variations in the O_2_ concentration in the large intestine, which in turn induce changes in the expression levels of HIF-1α, which has a role in the regulation of iron absorption by epithelial cells (Zheng et al., 2015; Henson and Phalak, 2017). Therefore, under pathological conditions, the increase in Fe^2+^ and other essential ions, such as Cu^2+^ or Zn^2+^, in the colon may induce the production of reactive oxygen species (ROS) affecting the anaerobic microbiota, thus leading to dysbiosis (Kohzadi et al., 2017; Pinho et al., 2021; Sohrabi et al., 2021).

Different approaches have been used to study the composition of the GITm (Namsolleck et al., 2004); colonic fermentation is the most frequently used technique to grow microorganisms from fecal samples, followed by 16S RNA gene metabarcoding analysis (this technique manages to cultivate 20-25 microbial species), metagenomic (shotgun) analysis, and fluorescence *in situ* hybridization (Blatchford et al., 2015; Vogtmann and Goedert, 2016; Gutiérrez-Sarmiento et al., 2020; Barros de Medeiros et al., 2021; Zhou et al., 2021). Quantitative imaging has also been used to study the spatial organization of gut microbiota (Earle et al., 2015). Despite these important advances, integral biochemical studies (culturomics, proteomics, ionomics, and kinetics) to explore the physiology of the gastrointestinal microbiota are still scarce. The co-culture of bacteria and archaea extracted from extremophile environments has been successfully implemented in our laboratory. Crenarchaeota and Euryarchaeota, as well as Thermotogales and Aquificae, from the Archaea and Bacteria domains, respectively, were mainly identified. Furthermore, with the development in the culture approach, metabolic interactions were identified between prokaryotes (Peña-Ocaña et al., 2022).

In this work, cultivation (culturomics) of anaerobic bacteria and methanogenic archaea present in fecal samples from healthy control (CR) and hepatoma-bearing rats (HR) was achieved to 1) understand the changes associated in microbial abundance and composition of GITm in an animal model of disease and 2) deepen in the physiology of the microbiota community. The biomass obtained was used for 16S rRNA gene metabarcoding and metabolic and enzymatic analyses. Culturomics indicated impairment of the antioxidant response and loss of microbial diversity in GITm as a consequence of bowel inflammation.

## Methods

### Ascitic AS30D cell proliferation

For the experiment, 3-month-old female Wistar rats of 200–250 g weight were injected intraperitoneally with 4 mL ascites hepatoma AS30D cells, while control rats were not injected with any innocuous solution as previously reported (Marín-Hernández et al., 2020). The animals were kept in cages with food and fresh water *ad libitum*, and 8 days after inoculation, the hepatoma was fully developed. Feces were collected just before animals were euthanized. All animals were manipulated according to the guidelines NOM-062-ZOO-1999 (Norma Oficial Mexicana) for the care and use of laboratory animals, and the project received approval from the Instituto Nacional de Cardiología (CICUAL) with number INC/CICUAL/006/2021.

### Growth conditions of anaerobic microbiota

The culture medium was prepared based on the high-salt medium designed for methanogens, modified for the cultivation of anaerobic extremophiles, and redesigned for anaerobic gastrointestinal microbiota cells (Sowers et al., 1993; Peña-Ocaña et al., 2022). Because one of the aims of this study was to evaluate the activity of enzymes involved in organic matter degradation of the cultured microbiota, insoluble fiber was not added; instead, specific concentrations of a wide range of substrates, such as carbohydrates (mono- and disaccharides), soluble triacetylglycerol (TAG), organic acids, and amino acids, were added. The concentrations of carbon sources used were 5 mM glycerol, 10 mM TAG, 10 mM glucose, 5 mM lactose, 1 mM maltose, 0.5 mM DL-lactate, and 0.1 % (w/v) amino acids from enzymatic digested casein. To promote the growth of methanogens, 50 mM acetate and 50 mM methanol were added; in addition, 100 mM NaCl and mineral and vitamin solutions were added (Peña-Ocaña et al., 2022). The culture medium was buffered with 10 mM NaHCO_3_ and 25 mM HEPES (final pH 6.8–7.0). Except for acetate, all stock solutions of carbon sources were sterilized by filtration under anaerobic conditions and were added before cell inoculation. The culture medium was bubbled in an anaerobic chamber (COY Instruments, OH, USA) filled with 80:15:5 ratio of N_2_, CO_2_, and H_2_, respectively. After 2 h, 0.5 mM cysteine was added to reach complete anaerobiosis, using resazurin 0.01% (w/V) as redox indicator. To promote the growth of sulfate-reducing bacteria, 0.05 mM FeSO_4_ was added, instead of sulfide. Afterward, 50 mL of the culture medium were poured in 100-mL bottles and sealed with Teflon stoppers and aluminum collars and sterilized by autoclaving. This medium was further named INC-07 (patent registration in process; file MX/u/2022/000169).

**Figure 1:**
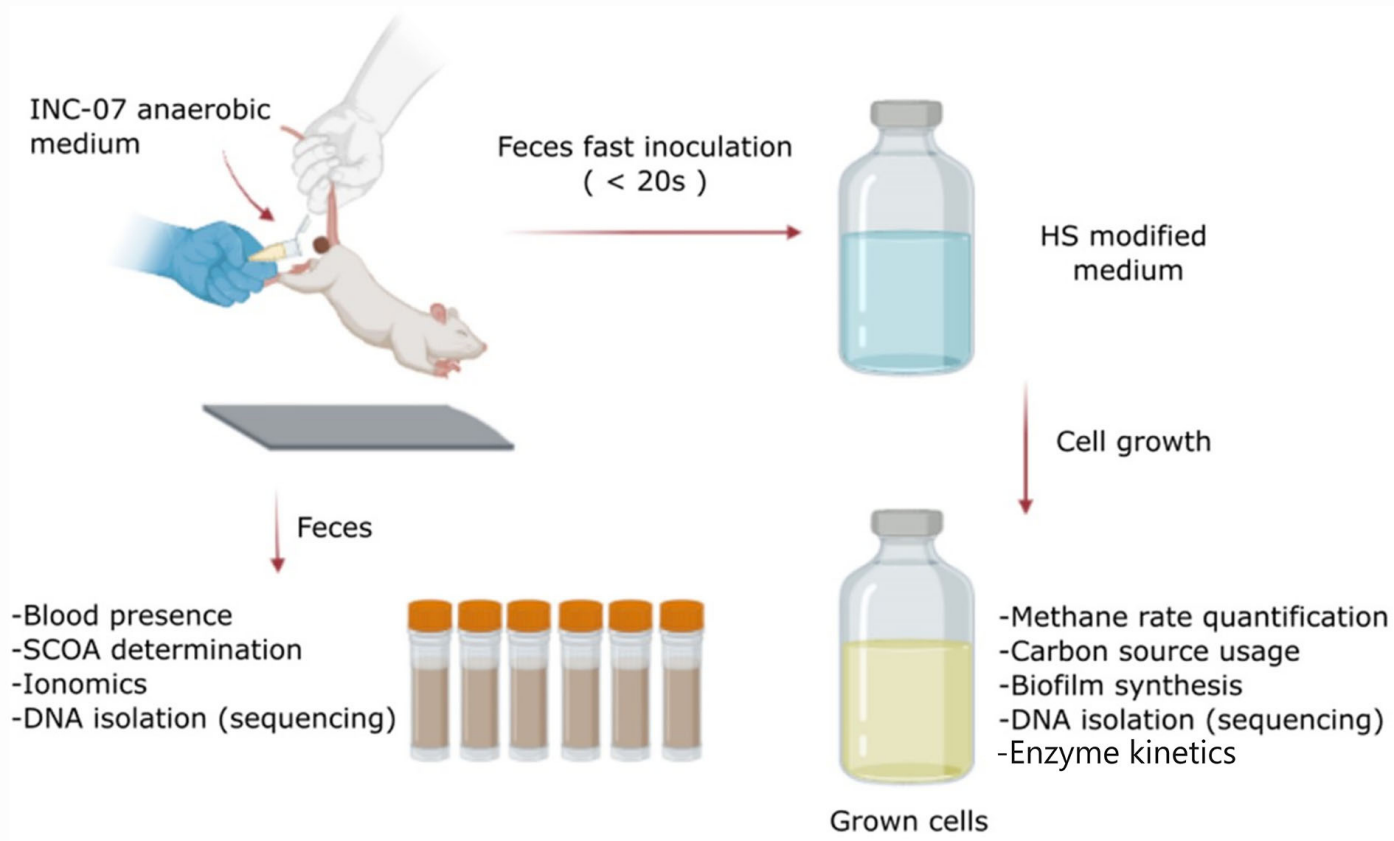
Scheme of the protocol followed in this study. Feces were collected from control and hepatoma rats just before euthanasia. The samples were used to determine changes in short-chain organic acids (SCOAs), ionomics, blood presence, and metagenomic analysis using 16S rRNA gene. On the other hand, a fraction of the feces was used for a quick inoculation in a medium designed for the growth of anaerobic microorganisms (INC-07 medium), to determine changes in abundance and variability of cultured species by analysis of 16S rRNA gene, biofilm synthesis, metabolites (SCOA production and carbon source consumption), enzyme kinetics, and ROS production. Created with BioRender.com.

To minimize the contact of the feces with air, feces were collected immediately after animal defecation, transferred to a sterile test tube containing 1 mL of INC-07 fresh medium, vortexed for a few seconds, and 0.2 mL of the vortexed sample was injected into culture bottles containing the INC-07 medium. The complete process lasted less than 20 s (Figure 1). It was determined that up to four subsequent transfer/passages using 2–3 mL inoculum, there was no loss in their ability to produce methane; therefore, the results showed here were from cultures with three passages. Microbiota cultures of control rats (CRC) and rats with hepatoma (HRC) were incubated in darkness at 37°C without shaking. Growth was monitored by measuring OD_600_; methane production was determined and used as a parameter of the complete oxidation of the carbon source consumed. As control, cultures of feces from rats before hepatoma inoculation were carried out, and no differences in methane production were attained between hepatoma and control rats; similarly, culture of feces from rats with hepatoma until 6 days after inoculation did not show differences in methane production with respect to control rats (not shown).

### Miscellaneous

Microbiological, biochemical, and 16S rRNA metabarcoding methodologies such as histopathological analysis carried out in this work are described in detail in the Supplementary material S1 text file.

### Statistical analysis

Alpha diversity based on ASVs was calculated using the vegan R studio package (version 1.2.1335, Inc., Boston, MA, USA) (Oksanen et al., 2020). To determine the microbial rarefaction curves, Chao1, ACE, Simpson, and Shannon indexes, the analysis was performed based on the mean values of the ASV data in triplicate. Graphic visualization of the 16S rRNA metabarcoding results was carried out with a relative abundance of the domain, phyla, and genus levels > 0.05% from the ASV count. For other culturomic results, Student's *t*-test was carried out. *P* < 0.05 was interpreted as significant.

## Results

### Effect of cancer cells on rat bowel integrity

**Figure 2:**
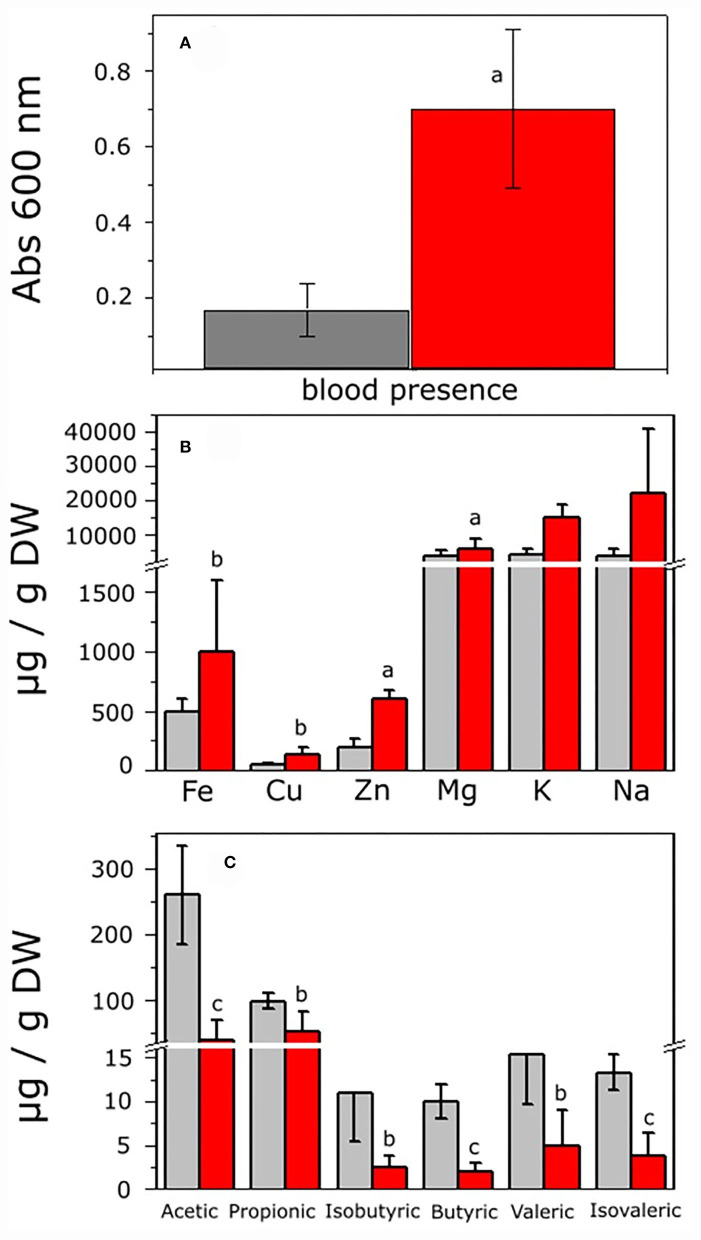
Analysis of feces from control and hepatoma-bearing rats. Feces were collected from control and rats with hepatoma and processed as indicated in the Methods section. **(A)** Detection of blood in feces as determined by the toluidine blue method. **(B)** Fe, Cu, Zn, Mg, K, and Na were determined by ICP-OES analysis. **(C)** SCOAs were extracted and acetic, propionic, isobutyric, butyric, valeric, and isovaleric acids quantified by gas chromatography, as described in the Supplementary materials. Values shown are the mean ± SD of six (for blood detection), five (for SCOA determination), and 12 (for ionomics) different rats, respectively. ^a^*P* < 0.01; ^b^*P* < 0.05; ^c^*P* < 0.001 *vs* feces from control rats.

To analyze the intestinal environment from the control and hepatoma rats in which microbiota developed before cultivation, histological analysis of the large intestine was carried out. The control rats (CR) showed normal intestine histology, flat mucosal surface with abundant vertically oriented Lieberkühn crypts, and well-defined muscular zone (Supplementary Figures S1A,C). By contrast, the rats with hepatoma (HR) showed superficial erosion of mucosa with severe atrophy and irregular microvilli and thickened irregular muscular zone (Supplementary Figures S1B,D). The highly stained regions observed after colloidal iron staining suggested an increment of total iron in the intestine of HR (Supplementary Figures S1C,D). The fecal occult blood test was positive in feces from HR, which was 4.2-fold higher than that of feces from the CR, as judged by the intense blue color from the complex formed by chromogen and hemoglobin (Figure 2A). Hence, oxygen (bound to hemoglobin) and iron may be present in the intestinal lumen of the HR due to tissue disruption.

### Essential ions and short-chain organic acids in feces

Disruption of the integrity of the epithelial barrier resulted in entrance of metals from blood to the lumen; the content of relevant pro-oxidant metals showed a significant increase of 2.3, 2.8, and 3 times higher for Fe, Cu, and Zn, respectively, in the same way, K increased 3.6 times, whereas Mg and Na remained roughly constant in HR vs. CR (Figure 2B). Hence, a potential oxidative stress condition might alter the GITm composition, leading to dysbiosis. To evaluate the latter, changes in the content of short-chain organic acids (SCOAs) produced by microbial metabolism as a parameter of microbiota diversity were determined in feces from the CR and HR. Acetic and propionic acids in the samples of CR were far more abundant than isobutyric, butyric, valeric, and isovaleric acids, but most of SCOAs showed a drastic decrement in samples from the HR (Figure 2C).

These data indicated that cancer provoked bowel inflammation and changes in Fe^2+^ levels in feces, possibly promoting a prooxidant environment on mostly the anaerobic microbiota cells present in the lumen of the intestine and a change in its composition in the diseased animals. However, further metabolic and biochemical analyses were impaired by the scarcity of the biological material found in the fecal samples, which made establishing a culturomic approach necessary to achieve such studies.

### Metabolism of the cultured microbiota cells

**Figure 3:**
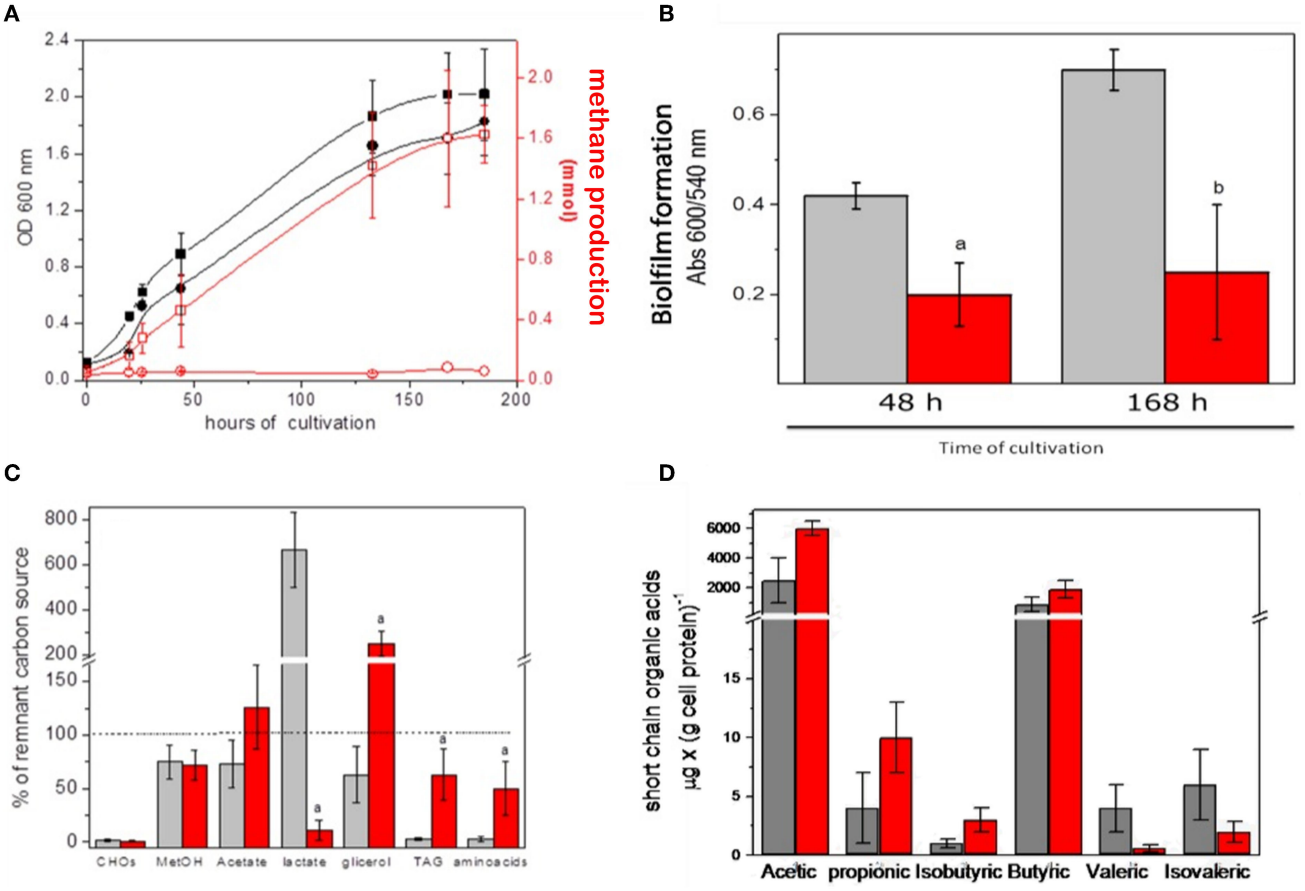
Gastrointestinal microbiota cultured in the INC-07 medium. **(A)** Growth curves and methane formation, **(B)** biofilm synthesis, **(C)** carbon source consumption, and **(D)** SCOAs excreted to the culture medium. CRC growth (black squares), methane production (open squares), HRC growth (red circles), and methane production (open circles) were determined throughout time. CRC are given in gray columns and HRC in red columns. Values are the mean ± SD of five different cultures. ^a^*P* < 0.001 vs. control samples. ^b^*P* < 0.05.

As described in Materials and Methods, the modified INC-07 medium was developed for culturing microbiota from fecal samples to obtain enough biological material for biochemical analyses *in vitro* (section Growth conditions of anaerobic microbiota). Figure 3 summarizes the results obtained from GITm grown in the INC-07 medium. No significant difference in cell growth was determined in microbiota of control rat fecal cultures (CRC) and hepatoma rat fecal cultures (HRC); however, methane production from GITm of HRC was completely diminished in comparison to CRC (Figure 3A). The CRC also produced 2.5–3 times more biofilm than HRC (Figure 3B). Lower biofilm synthesis in HRC suggested that the GITm of the rats suffered dysbiosis status due to cancer invasion.

Changes in CO_2_ production/consumption were also found (not shown), suggesting active cell metabolism. The preference of CRC for carbon source consumption was carbohydrates equal to TAG and amino acids (90%) > methanol, acetate, and glycerol (25–40%) and overproduction of lactate; in turn, HRC consumed carbohydrates > lactate (90 %) > methanol, TAG, and amino acids (30–50%) and overproduced acetate and glycerol (Figure 3C). Metabolism of the GITm cultured in the INC-07 medium also produced SCOAs as previously determined in the fecal samples, although at different levels. Acetic acid was by far the most abundant acid determined in the CRC and HRC samples, but mostly due to external addition (as carbon source); the butyric acid content (which is probably the most important short-chain organic acid involved for human wellbeing) was abundant in both types of cultures. In turn, propionic and isobutyric acids were higher in the HRC than in the CRC, but valeric and isovaleric acids were higher in CRC (Figure 3D). These data suggested different metabolic performance due to diversity and abundance of cultured GITm between the healthy control and rats with cancer.

High yield of biomass allowed determination of enzyme activities involved in carbon catabolism in cellular fractions from both types of cultures (Table 1). Significantly higher enzyme activities of lipases and glycerol dehydrogenase were found in the CRC, which correlated with higher consumption of TAG and glycerol in these cultures. Activities of hexokinase (glucokinase), maltase (alpha-glucosidase), and lactase (beta-galactosidase) were similar between the CRC and HRC, which correlated with the efficient utilization of carbohydrates in both cultures. Acetate kinase, which catalyzes the reversible reaction (production of acetate and ATP in bacteria and acetyl-P and ADP in acetoclastic methanogenesis in archaea), was very active in both cultures. In turn, the CRC and HRC showed high activity of soluble NAD^+^-lactate dehydrogenase but lower activity of membrane-bound NAD^+^-independent lactate dehydrogenase. ALAT and ASAT transaminases, key enzymes in amino acid metabolism, were also present, with remarkably 15 times higher ALAT activity in the soluble fraction from the HRC.

**Table 1:** Enzyme activities related to carbon sources consumption in GITm.

	**Rat control cultured GITm**	**Rat with hepatome cultured GITm**
Enzyme	Specific activity nmol (min × mg protein)^−1^	
acetate kinase	510 ± 246 (5)	357 ± 248 (5)
aspartate transaminase	23 ± 2 (4)	29 ± 7 (5)
alanine transaminase	11 ± 2 (4)	151 ± 40^a^ (6)
hexokinase	37 ± 14 (5)	63 ± 43 (5)
maltase	326 ± 171 (5)	186 ± 106 (5)
lactase	306 ± 249 (5)	243 ± 98 (5)
lipases	723 ± 260(4)	24 ± 9 ^a^ (4)
NAD^+^-glycerol dehydrogenase	13 ± 8 (6)	2 ± 1.5^b^ (6)
NAD^+^-lactate dehydrogenase	1373 ± 514 (6)	785 ± 342 (6)
Membrane-bound lactate dehydrogenase	23 ± 2 (6)	8 ± 6^b^ (6)

Activities were determined in cytosolic or membrane fractions as described in the Methods section. Values shown are the mean ± SD of the number of independent cultures indicated within parentheses. ^a^*P* <0.01; ^b^*P* < 0.05 vs. control GITm.

### Oxidative stress and antioxidant enzymes

The occurrence of blood in the feces of HR suggested the presence of O_2_ and extra Fe in the lumen of the gastrointestinal tract, which may disturb the otherwise strict anaerobic environment. Indeed, when CRC microbiota were incubated for 3 and 7 days in a combination of air (1%) plus a mixture of ions (Fe^2+^, Cu^2+^, Zn^2+^, and K^+^), simulating the conditions found in the bowel of the HR, methane production was strongly affected (Supplementary Figure S2).

Furthermore, the rate of ROS production was determined in cell suspensions, showing that cells of the HRC produced two times more ROS than cells from the CRC; GR activity was also significantly higher in HRC cells, whereas GPx activity was similar respect to CRC cells (Table 2). However, activities of the NADPH-producing enzymes (IDH, ME, and Glc6PDH) were strongly affected in HRC cells, which may impede the efficient provision of reducing equivalents for ROS detoxification (Table 2).

**Table 2:** ROS production and antioxidant enzymes in rat microbiota cultures.

	**Control Rat GITm**	**Rat with hepatoma GITm**
**ROS**	pmoles ROS _produced_ (min × mg protein)^−1^	
production rate	150 ± 50	300 ± 42^a^
**Enzyme activity**	nmoles (min × mg protein)^−1^	
GSH reductase	22 ± 8	42 ± 6^a^
GSH peroxidase	50 ± 11	60 ± 3
NADP^+^- IDH	43 ± 11	3 ± 1^b^
NADP^+^-ME	7 ± 2	1 ± 1^b^
NADP^+^-Glc6PDH	8 ± 1	3 ± 1^b^

ROS production was determined in cells under anaerobic conditions, and enzyme activities were determined in cytosolic fractions as described in the Methods section. Values shown are the mean ± SD of four independent batches. ^a^*P* < 0.05; ^b^*P* < 0.001 vs. control.

### Metabarcoding analysis

**Figure 4:**
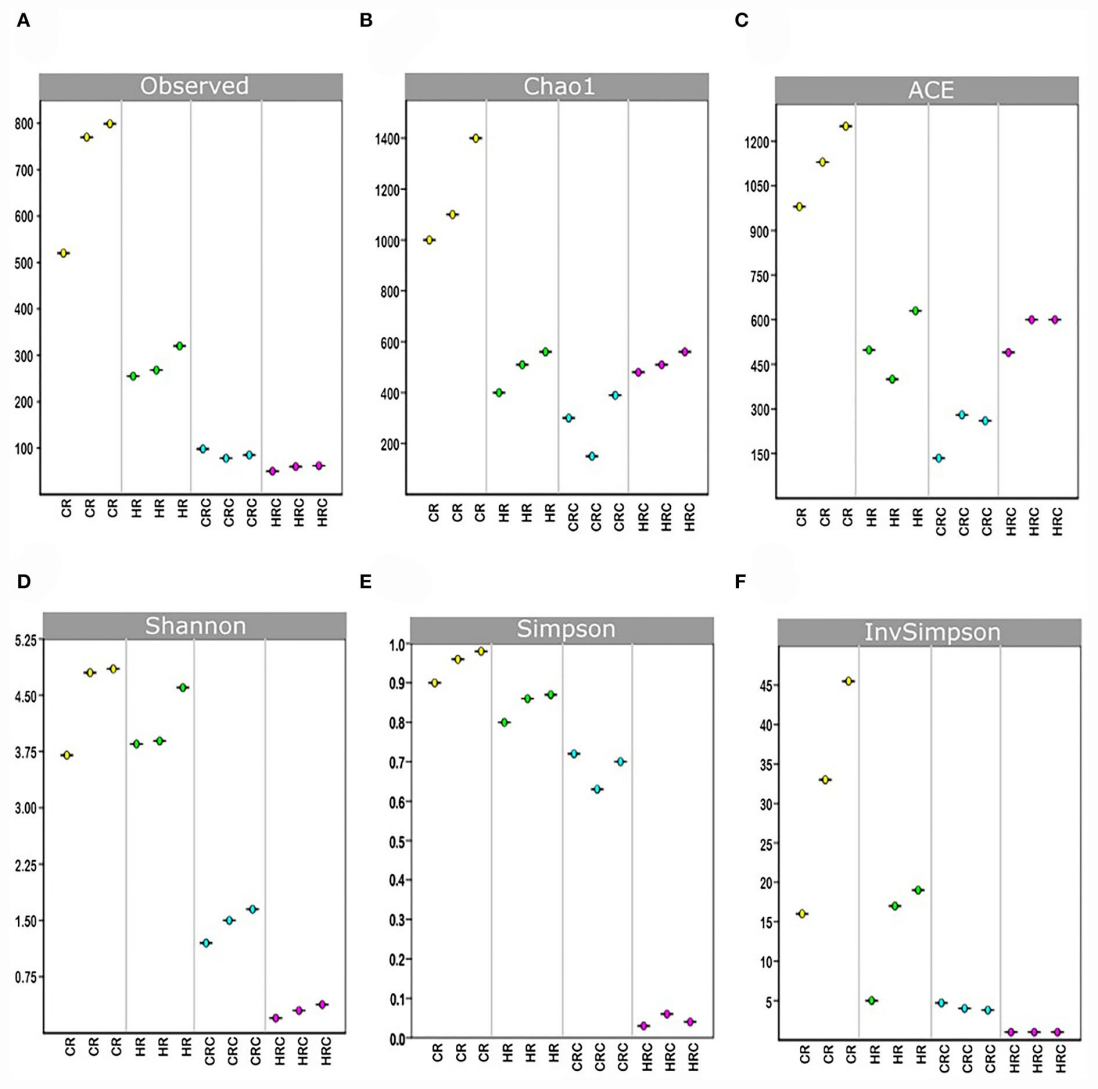
Indexes of richness. Observed **(A)**, Chao1 **(B)**, and ACE **(C)** and indexes of uniformity Shannon **(D)**, Simpson **(E)**, and 1/Simpson **(F)**, were plotted based on the complete ASV data set determined in fecal samples from control (CR) and hepatoma (HR) rats, and their respective microbiota cultures CRC and HRC. *P* < 0.01, ANOVA unidirectional and the LSD multiple range test.

Yields of DNA extracted showed non-significant differences between the CRC and HRC, whereas the amount of DNA recovered was 2–3-times higher in feces from CR than in feces from HR (*n* = 3, *P* < 0.01). In CR feces and CRC (after three cultivation transfers), 1,167 and 236 unique amplicon sequence variants (ASVs) were identified at 0.05% relative abundance, respectively, whereas in HR feces and HRC, 510 and 500 ASVs were identified, respectively. Relative abundances at different values are shown in Table A1. The ASV CR/CRC ratio was 4.92, and the HR/HRC ratio was 1.02. The number of ASVs of high-quality sequences and adequate length are shown in the rarefaction curves (Supplementary Figure S3). Asymptotes of CRC, HRC, and HR curves indicated a satisfactory identification of GITm, whereas non-saturating curves from CR samples indicate an incomplete GITm representation, which may improve with higher sequencing steps. Data supported the occurrence of taxonomic changes induced by the growth of the hepatoma cells. Although ASVs can detect small changes in some regions of 16S rRNA, they may also produce technical errors, especially when data quality is insufficient or the set of data is too small (Joos et al., 2020). To avoid over-interpretation of identified ASVs, the alpha diversity estimators, which describe the number of different species that should be found in each sample, were emphasized (Calle, 2019) (Figure 4). The observed index showed higher diversity of the microbial species in feces (CR and HR) than in samples from cultures (CRC and HRC) (*P* < 0.01). Absolute values for the CR and CRC were 696 ± 165 and 98 ± 2, respectively, and for HR and HRC, 278 ± 27and 61 ± 6, respectively (Figure 4A). CR showed a Chao1 index value of 1,217 ± 225, whereas CRC and HRC, as well as HR, showed a lower Chao1 index values, ranging from 300 to 500; a higher value suggested the presence of a higher number of rare microorganisms or with small representation. In agreement with the ACE, Simpson, and InvSimpson, the Shannon–Weaver index (H) indicated that the GITm analyzed from feces from the CR and HR is highly diverse (H = 4.5 ± 0.2 and H =3.63 ± 0.11, respectively); in turn, CRC microbiota was more diverse (H = 1.46 ± 0.15) than HRC microbiota (H = 0.56 ± 0.3). Therefore, after carefully revision, the final depuration of ASVs from CRC and HRC resulted in the identification of 98 and 61 different species (bacteria and archaea), respectively, as many ASVs were identified as the same species. In the same way, to estimate the overall difference of the microbial communities, we evaluated the beta diversity using the Jaccard index (Hu et al., 2018). The diversity identified in HR was 40% lower with respect to CR, and 79% lower for HRC with respect to CRC. This decrement in diversity in HRC could be a sign of the proliferation of the hepatoma cells affecting the intestinal microbiota. In turn, CRC diversity was 67% lower with respect to CR, as HRC was 58% lower as for HR. The latter is indicative of the efficiency of the INC-07 growth medium.

**Figure 5:**
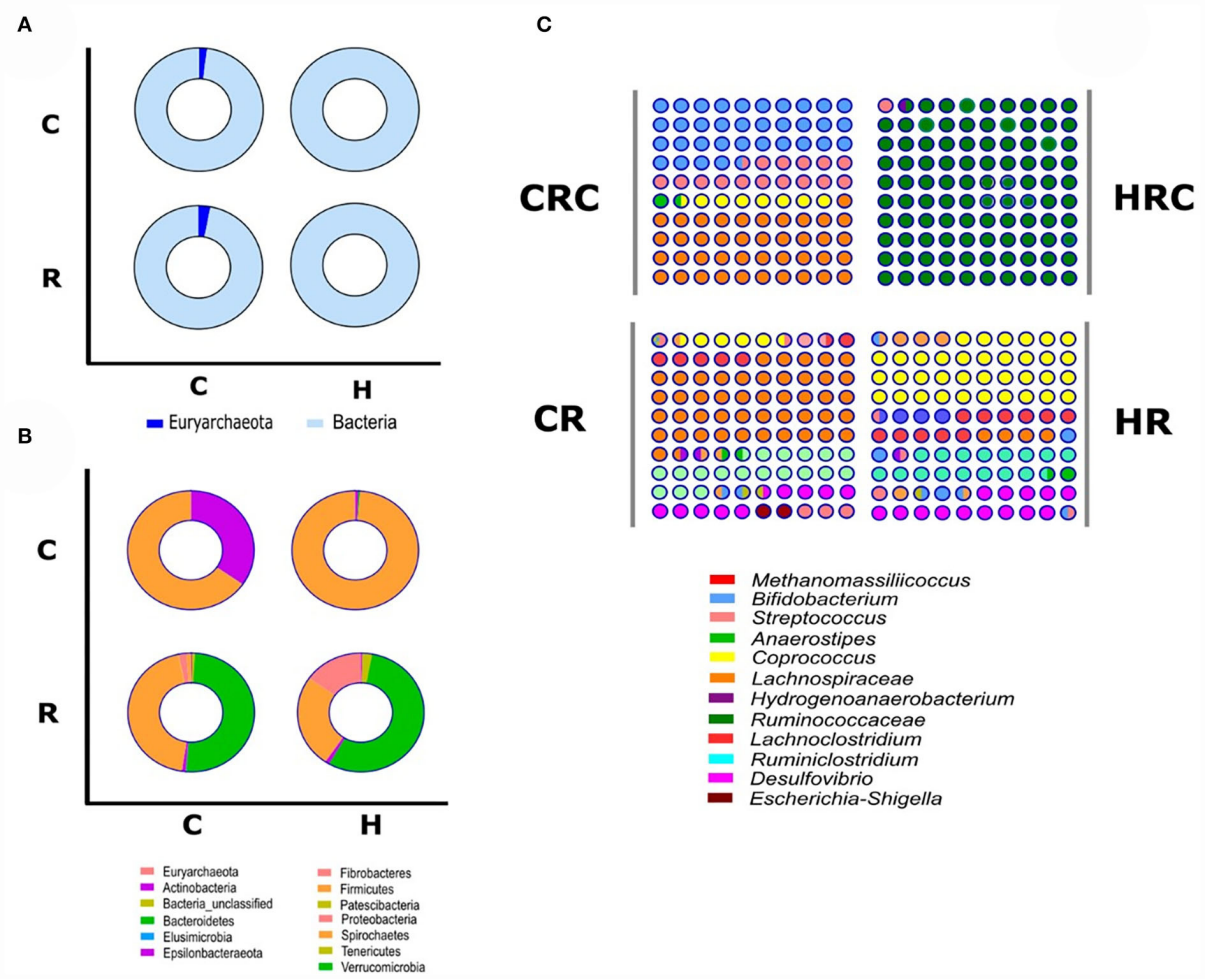
Microbial diversity in feces and culture samples. Microbiota derived from feces of control rats (CR), rats with hepatoma (HR), and direct from rats (R) and cultures (C). Microorganisms were analyzed by Domain **(A)** Euryarchaeota (dark blue) and Bacteria (cyan), by phylum **(B)**, and by genus **(C)**. Only microorganisms with a relative abundance >0.05 % were considered and evaluated by 16S rRNA analysis.

The use of the V3-V4 regions of the 16S rRNA gene to perform the taxonomic assignment of prokaryotes allowed for the simultaneous identification of bacteria and archaea in feces and its cultures. In control GITm (CR and CRC), archaea was represented by the phylum *Euryarchaeota*, with 2–4% abundance; by contrast, in HR and HRC, the absence of archaea and methane was evident (Figure 5A). *Bacteria* and *Archaea* were identified, with 39 genera distributed in 13 phyla (Figure 5B); the more abundant microbes in CR feces were Bacteroidetes 53% and Firmicutes 42%, and there was marginal presence of Actinobacteria, Fibrobacteres, and Spyrochetes (5%). The phyla mainly represented by the next genera (Figure 5C) were *Prevotella* (38.14%), *Alloprevotella* (5.65%), *Lactobacillus* (15%), *Lachnospiraceae* (7.7%), *Roseburia* (1.35%), *Butyricicoccus* (1%), and *Ruminococcus* (7%); two methanogenic archaea were identified: *Methanobrevibacter* and *Methanomassiliicoccus* (4%). In HR feces, phyla identified were 61% Bacteroidetes, 19% Firmicutes, 12% Proteobacteria, and 5% Patescibacteria, Actinobacteria, and Epsilonbacteraeota. The predominant genera identified were *Bacteroides* (28.7 %), *Lachnospiraceae* (10.7%), *Alloprevotella* (8.7%), *Prevotella* (3.35%), *Tannerellaceae*_*unclassified* (13.22%), *Helicobacter* (1.26%), *Ruminococcus* (3.8%), and *Salmonella*-*Shigella* (5.8 %). Data obtained from the cultures were as follows: in the CRC samples, phyla identified were Firmicutes (65%), *Lachnospiraceae* (41%), *Streptococcus* (15%), *Coprococcus* (7.57%), and Actinobacteria (35%), which was mainly represented by *Bifidobacterium* (34.6%). In HRC samples, Firmicutes represented mainly by *Streptococcus* (98%) prevailed, and Bacteroidetes, Actinobacteria, *Bifidobacterium* (0.78%), and *Prevotella* (0.23%) were the most abundant.

**Figure 6:**
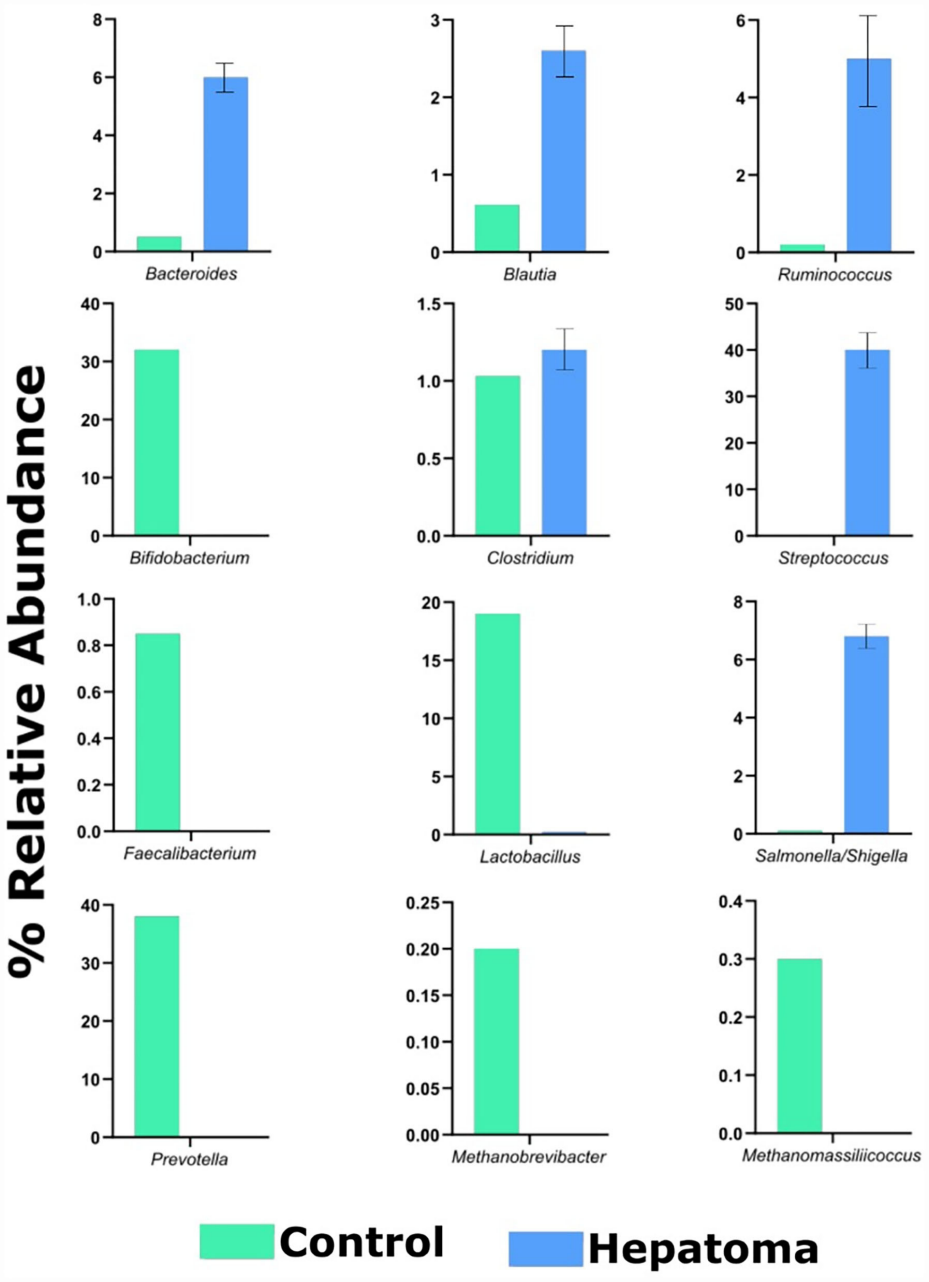
Bar plot of relative abundance of cultured microorganisms with clinical relevance. Analysis of relative abundances of beneficial and pathogenic ASVs was carried out in CRC (green columns) and HRC (blue columns). All species showed significance of *P* < 0.001 vs. control samples.

Comparison of the control and hepatoma conditions revealed that control microbiota showed beneficial prokaryotes such as *Bifidobacterium, Faecalibacterium, Prevotella, Lactobacillus, Methanobrevibacter*, and *Methanomassiliicoccus* of clinical relevance; therefore, they may be proposed as marker organisms of a healthy microbiota. By contrast, in the dysbiotic HR microbiota, their abundance was much lower, allowing bacteria considered potentially pathogenic such as *Salmonella, Shigella*, and *Streptococcus* to proliferate (Figure 6).

### Prediction of the metabolic potential of the GITm in control and hepatoma rats

The microbial community abundance was used to predict the metabolic changes of the GITm due to the presence of the hepatoma in rats. For this purpose, a phylogenetic investigation of communities by reconstruction of unobserved states (PICRUSt) was conducted. The predicted pathways in control conditions (CR and CRC), as well as in the samples from the rats with hepatoma (HR and HRC), were grouped in archaea and bacteria (Supplementary Figure S4).

A total of 131 pathways from the Kyoto Encyclopedia of Genes and Genomes (KEGG) from the 16S rRNA functional ortholog sequences (KO) were analyzed. Fifty nine pathways of KO level 3 with a value higher to 1% of abundance were found, of these 38 pathways were gathered in pathways of KO level 2. These abundant KOs represented 60% of the total processes predicted, and they corresponded to the KOs for thiamine metabolism, Krebs cycle, and metabolism of amino acids in archaea, whereas for bacteria, the most abundant processes were for fatty acids, carbohydrates, and amino acid metabolism.

Finally, the canonical correspondence analysis (CCA) was carried out to evaluate the degree of correlation between metabolic capabilities established (consumption of carbon sources and production of short-chain organic acids, methane and CO_2_) and microbial communities found (most abundant taxonomy). A 69% relationship between microbial species and metabolic variables with a permutability analysis of 999 and a Monte Carlo permutation linear relationship test of 0.089 was determined. The two-dimensional map of axes 1 and 3 showed a strong relationship between the biological models of healthy and diseased states.

Hence, *Faecalibacterium, Bifidobacterium, Methanomassiliicoccus, Methanobrevibacter*, among others were identified in the healthy GITm, as well as high amounts of SCOAs such as butyric, valeric, and propionic acids and their isoforms. The INC-07 culture system favored methanogenic metabolism, observing a strong correlation with the production of methane and higher production of acetate and CO_2_ (Supplementary Figure S5). On the other hand, the HRC correlated with low consumption of carbohydrates and amino acids and a high consumption of the TAG available in the culture medium. Remarkably, undesirable bacteria such as *Enterobacteria* (*Salmonella/Shigella*), *Helicobacter, Enterococcus*, and *Streptococcus* in the disease model were abundant.

## Discussion

### Changes in the GITm composition induced by bowel inflammation in rats harboring hepatoma AS30D

The AS30D ascites tumor cells are of epithelial origin, and the ascitic fluid becomes increasingly hemorrhagic after 5 days of tumor growth (Smith et al., 1970). In this work, rats after 8 days of hepatoma developed severe bowel inflammation, presence of blood in feces (with concomitant increase of O_2_), and an increase in pro-oxidants Fe, Cu, and Zn, as well as K, in feces. These events induced dysbiosis in the gastrointestinal tract of the rats due to the sensitivity of anaerobic microorganisms to oxidative conditions. The abundance of *Bifidobacterium, Faecalibacterium, Prevotella, Lactobacillus, Methanobrevibacter*, and *Methanomassiliicoccus* was highly present the feces from HR presumably due to oxidative stress. These prokaryotes are important in the production of acetate and propionate, which can be used by mitochondria from the intestinal epithelium cells (Dugas et al., 2018; Jackson and Theiss, 2020). In the lumen of the intestine, hyperbaric O_2_ alters the gut microbiota composition, mainly affecting the strict anaerobes, which have been cataloged as catalase-positive bacteria, although enzymatic activity was not determined (Albenberg et al., 2014).

### Effect of oxygen on GITm

Difficulties in co-cultivation of bacteria and archaea are the main limitation for further experimental approaches such as proteomic, metabolomic, kinetic, and fluxomic analyses, as well as metagenomics. These studies are required to understand the subtle balance between nutrient-consuming microorganisms and benefit producers; the disruption of this balance promotes dysbiosis (Albenberg et al., 2014; Zheng et al., 2015; Henson and Phalak, 2017; Kapoore et al., 2021; Xu et al., 2021; Zhang et al., 2021). The design of cultures may help evaluate the complex interaction between different microorganisms present in microbiota and the effect of external stressors such as oxygen (Smith et al., 2019). It has been reported that 1 μM O_2_ can induce inflammatory bowel disease in rats, resulting in a decrease in *Faecalibacterium prausnitzii* in the gut and a large increase in *E. coli*. The authors concluded that dysbiosis was consistent with disease progression and could be qualitatively predicted solely based on metabolic differences between the species (Henson and Phalak, 2017). However, this conclusion was based by analyzing a community of only three species. Studies on more complex microbiota communities are restricted due to complications in cultivation *in vitro*. A strategy to circumvent this limitation was one of the main objectives of the present study, which was successfully achieved by the design of the INC-07 medium, which allows growth of bacteria and archaea. Our results demonstrated the high susceptibility of the microbial network to an O_2_ pulse plus a mixture of Fe, Cu, and Zn, which dramatically decreased methane synthesis in control cultures.

It is reported that the presence of blood in feces increases the abundance of *Bacteroides uniformis, Collinsella aerofaciens, Eggerthella lenta, and Clostridium symbiosum*, while decreases the abundance of *Prevotella copri, Coprococcuseu tactus and catus, Faecalibacterium prausnitzii, Roseburia faecis, Blautia obeum, Gemmiger formicilis, and Clostridium celatum* (Chénard et al., 2020).

### Metabolic properties of cultured gastrointestinal microbiota

Methane production detected from GITm cultured in the INC-07 medium and the identification by metabarcoding of hydrogenotrophic *Methanobrevibacter* and methylotrophic *Methanomassiliicoccus* led to two main interpretations: i) H_2_ production by bacteria-metabolizing carbohydrates and lactate, and ii) sulfide production by sulfate-reducing bacteria since they thrived and produced enough sulfide for the successful growth of methanogens (Smith et al., 2019). The presence of methylotrophic methanogens may be relevant for the consumption of cardiotoxic methylamines from the gut or methanol present in the INC-07 medium, but H_2_ is required for their metabolism (Kurth et al., 2020). The successful co-culture of bacteria with archaea demonstrated the importance of methanogens in a healthy GITm and that their absence may generate dysbiosis (Djemai et al., 2021; Miller et al., 2021). Hence, secondary metabolites produced by the complete oxidation of the carbon source consumed were H_2_, CO_2_, sulfide, methane, and SCOAs.

The presence, induction, and activities of enzymes related to the catabolism of nutrients and xenobiotics and their changes due to illness have been determined not only in the serum, liver, and intestine from human but also in gastrointestinal microbiota. Acetate kinase, sucrase, lactase, and lipase have been extensively studied (Kushkevych, 2014; Lin et al., 2022; Zeinali et al., 2022). On the other hand, enzymes related to protection against oxidative stress have also been determined in microbiota studies (Yu et al., 2021; Panigrahi et al., 2022; Zhu et al., 2022). From GITm cultured in the INC-07 medium, it was feasible to prepare cytosolic and membrane fractions to identify key components of the enzymatic machinery involved in the degradation of carbon sources added, as well as those that contend against oxidative stress. Differences in catabolic enzymes and in the capacity of NADPH production, indispensable to maintain reduced GSH (or other thiol-containing molecules) to contend with ROS, were lower in dysbiotic GITm.

Butyrate-producing organisms proliferate in the INC-07 medium. This metabolite has been involved in *i*) the synthesis of some interleukins (IL6 and IL10) by healthy intestinal tissues; *ii*) the regulation of the levels of the brain-derived neurotrophic factor (BDNF), which plays a vital role in neuronal survival and growth; and *iii*) the synthesis of gamma-aminobutyric acid, which is transported to the brain to anxiety-like behavior (Romo-Araiza et al., 2018; Hao et al., 2019; Silva et al., 2020). Production of SCOAs (preferentially butyrate) was low in fecal samples of rats with hepatoma (HR), probably due to an imbalance in the abundance between butyrate-producing/consuming microorganisms. The family of *Lachnospiraceae* belonging to the *Clostridiales* order shows metabolic capacities for polysaccharides and short-chain alcohol and acid fermentation, such as acetate and butyrate. It has been reported that high levels of butyrate are related to a protective effect against colon cancer incidence (Meehan and Beiko, 2014; Ai et al., 2019). A low content of SCOAs in feces of HR might be related to growth of AS30D cells since it has been demonstrated that acetate and propionate are consumed by cancer cells (Rodríguez-Enríquez et al., 2021). Probably, many other enzymes and metabolic pathways can be studied in detail with these cultures without the necessity of purification from feces, which facilitates further experimental approaches.

Hepatoma induced a decrement of beneficial bacteria such as *Prevotella, Faecalibacterium, Lachnospiraceae, Bifidobacterium*, and *Lactobacillus;* these organisms have been proposed as essential in the maintenance of a healthy microbiota because they can improve glucose metabolism and digestion of complex fibers, and some of these represent biomarkers of a healthy intestine and are related to the modulation of the immune system (Meance et al., 2003; Kovatcheva-Datchary et al., 2015; Vuillermin et al., 2020). These organisms were substituted by genera *Bacteroides, Parabacteroides* (*Tannerellaceae*) *Helicobacter, Streptococcus*, and *Salmonella-Shigella* in microbiota of HR. Most of these bacteria are related to dysbiosis, infectious diseases, and cancer (Gao et al., 2018; Xie et al., 2019; Guo et al., 2020; Sha et al., 2020; Devi et al., 2021; Cao et al., 2022).

### Metabarcoding analysis indicated changes in microbial diversity under dysbiosis

According to different authors, the core of the gastrointestinal microbiota is constituted preferentially by *Firmicutes, Bacteroidetes, Proteobacteria*, and *Actinobacteria*, as well as other taxonomic divisions of minor abundance (Duda-Chodak et al., 2015). Interestingly, Kim et al. (2020) analyzed fecal samples from ~900 persons from Korea, where archaea represented 10% of all microorganisms with most members of the methane-producing archaea. In this work, in the GITm of healthy rats, *Archaea* of the *Euryarchaeota* phylum represented by the genera *Methanomassiliicoccus* and *Methanobrevibacter* constituted 2–4%. The hydrogenotrophic *Methanobrevibacter* is the main methane producer found in the digestive tract of humans with its colonization starting after birth, and it is also abundant in the gut microbiota in athletes, suggesting that in rat and human, methanogens are present in a presumably healthy individuals (Grine et al., 2017; Scheiman et al., 2019; Wosinska et al., 2019; Mohr et al., 2020).

Information about the relevance of archaea in the gastrointestinal microbial network is scarce. Most microbiota studies are focused on bacteria, and although some have begun the search and identity of eukaryotic microorganisms (i.e., fungi), the group of archaea is still largely ignored mainly due to technical difficulties in their experimental handling, due to their lower percentage than other groups, and because no archaeal pathogens have been identified (Pausan et al., 2019). Nevertheless, methane production and archaeal presence only in healthy rats suggested an important and unknown role for a healthy microbiota. Therefore, in the microbiota cultured in INC-07 medium developed here, methane is proposed as a biomarker of intestinal health. Parkar et al. (2021) in an *in vitro* hindgut model reported 11 microbial genera at the same percentage that it was used in this work (0.05%), whereas in the INC-07 system, 16 genera corresponding to 98–150 species were identified. When the relative abundance at 0.1% was carried out, 54 and 80 species were identified in the INC-07 medium for HRC and CRC, respectively, whereas Blatchford et al. (2015) reported 23 species in the colonic fermentation *in vitro*. Therefore, INC-07 medium was highly effective when comparing the recovered diversity indexes with other previously reported results obtained by *in vitro* colonic fermentation.

## Conclusion

The INC-07 medium was a successful alternative for growing a huge diversity of microbial components from the gastrointestinal tract (H_2_ producers/consumers, sulfate reducers, methanogens), including those of clinical relevance. This cultivation system allowed for the validation (or contrasting) of predictive modeling and inferences in the metabolic dynamics derived from the use of the 16S rRNA. Thanks to the wide diversity of microorganisms cultured, it was possible to carry out metabolic and kinetic analyses in isolated cytosolic fractions. This study provides a new approach to evaluate the relationship between diseases and the gastrointestinal microbiota and the development of strategies, which allow for the restoration of a healthy microbiota. In the same way, this cultivation system may be highly effective for studying the effects of factors that disturb the delicate equilibrium of the gastrointestinal microbiota.

## Data availability statement

The datasets presented in this study can be found online at https://www.ncbi.nlm.nih.gov/ with the accession number KFUR00000000.

## Ethics statement

The animal study was reviewed and approved by the guidelines NOM-062-ZOO-1999 (Norma Oficial Mexicana) for the care and use of laboratory animals and the project received approval from Instituto Nacional de Cardiología CICUAL with number INC/CICUAL/006/2021.

## Author contributions

RJ-C: conceptualization and methodology. RJ-C, ÁM-H, TM, and RG-C: resources. RJ-C, BA-PO, YH, MS-F, IP-T, WG-S, LH-E, ÁM-H, and RS-T: investigation. R-JC, BA-PO, JS-RZ, ÁM-H, ES, IP-T, RS-T, RG-C, LH-E, and WG-S: formal analysis and data interpretation. RJ-C, BA-PO, YH, MS-F, JS-RZ, ÁM-H, RG-C, RS-T, and ES: original draft. RJ-C, BA-PO, JS-RZ, TM, and ES: writing—review and editing. All authors confirm that the work reported in this article and have approved the final submitted version.

## Funding

This work was supported by grants [CPC2022 #320299 (BA-PO) and CONACYT CB 2017–2018 # A1-S-8530 and PAPITT UNAM # IN214218 (RG-C) and # A1-S-40481 (ÁM-H)] from CONACyT, Mexico, and by the Sakura Science Exchange Program, Japan Science and Technology Agency [# P2020A0228092 (TM)]. MS-F was supported by CONACYT-Mexico fellowship (No. 1084917) from the program 000237-Maestría en Ciencias Bioquímicas, Facultad de Química, UNAM.

## Conflict of interest

The authors declare that the research was conducted in the absence of any commercial or financial relationships that could be construed as a potential conflict of interest.

## Publisher's note

All claims expressed in this article are solely those of the authors and do not necessarily represent those of their affiliated organizations, or those of the publisher, the editors and the reviewers. Any product that may be evaluated in this article, or claim that may be made by its manufacturer, is not guaranteed or endorsed by the publisher.

## Supplementary material

Supplementary Figure S1 Histological section of control **(A,C)** and with hepatoma **(B,D)**, rat colon stained with hematoxylin-eosin **(A,B)** or with colloidal iron **(C,D)**. Image analysis was made at 20X. Figure is representative of a total of 6 rat samples.

Supplementary Figure S2 Effect of stressors in methane production in cultured microbiota. Control microbiota was cultured in the absence (control, white column) or in the presence of a metal mixture (0.5 mM FeSO_4_, 0.1 mM CuSO_4_, 1 mM ZnCl_2_ and 50 mM KCl) plus 1% (v/v) of O_2_. Methane production was determined at day 3 (light gray column) and day 7 (gray column) of growth. Values shown are the mean ± SD of 3 independent cultures. ^a^ *P* <0.001 vs. control cultures.

Supplementary Figure S3 Rarefaction plot of amplicon sequences variants (ASVs) from CRC, CR, HRC, and HR. The *x* axis indicates the number of sequences and *y* axis indicates the observed microorganisms in ASVs. Each curve represents a different sample replicate.

Supplementary Figure S4 Heat map of metabolic function predicted by archaea **(A)** and bacteria **(B)** based on community phylogenetic research by reconstructing Unobserved State Analysis (PICRUSt) and the Kyoto Encyclopedia of Genes and Genomes (KEGG). The rows indicate the KEGG level 2 pathway (> 1% abundance) and the columns show the hierarchical groups of the *in vivo* control model (CR), the AS30D hepatoma *in vivo* model (HR), the control culture (CRC) and the culture of the microbiota of rats with hepatoma (HRC).

Supplementary Figure S5 Canonical correspondence analysis (CCA) of gut microbial communities and metabolic parameters ASV with relative abundance higher than 0.01% and which is also a marker for microorganisms of intestinal metabolism were applied for CCA, as environmental conditions, the metabolic variables of organic acids, CO_2_ and methane production, and carbon sources consumption were considered.

## Abbreviations

SCOAs: short-chain organic acids
CR: control rats
HR: rats with hepatoma
CRC: fecal cultures of control rats
HRC: fecal cultures of rats with hepatoma
GITm: gastrointestinal microbiota
TAG: triacetylglycerol
GPX: glutathione peroxidase
GR: glutathione reductase
NADP^+^-IDH: NADP^+^-isocitrate dehydrogenase
NADP^+^-ME: NADP^+^-malic enzyme
NADP^+^-Glc6PDH: NADP^+^-glucose-6-phosphate dehydrogenase
ROS: reactive oxygen species
RNS: reactive nitrogen species.

## Appendix

**Table A1:** Illumina MiSeq sequencing summary and amplicon sequencing variants (ASVs) identified per treatment.

**Sample**	**DNA** **(ng/mg sample)**	**Ratio 260/280**	**Paired-end reads**	**Number of ASVs** **0.001% abundance**	**Number of ASVs** **0.05% abundance**	**Number of ASVs** **0.1%** **abundance**
CRC	51 ± 10^a^	1.76 ± 0.10	13,656 ± 219^a,b^	7,714 ± 58^A^	237 ± 49^A^	80 ± 18^A^
HRC	42 ± 28^a^	1.66 ± 0.11	11,628 ± 862^b^	7,880 ± 9^A^	500 ± 40^B^	54 ± 25^A^
CR	68 ± 27^b^	1.76 ± 0.03	14,919 ± 1552^a^	5,514 ± 565^C^	1167 ± 153^C^	662 ± 128^C^
HR	20 ± 4.7^c^	1.78 ± 0.02	12,135 ± 951^b^	7,194 ± 142^B^	510 ± 26^B^	225 ± 26^B^

Values shown are the mean ± SD of 3 independent samples: CRC, microbiota cultures of control rats; HRC, microbiota cultures of rats with hepatoma; CR, GITm of control rats; HR, GITm of rats with hepatoma.^a, b, c^*P* ≤ 0.01 using ANOVA Fisher test (LSD) DNA vs. reads per sample.^A, B, C^*P* ≤ 0.01 in sequences vs. ASVs.
