# Supplementary figures and images for: Cultivation of gastrointestinal microbiota in a new growth system revealed dysbiosis and metabolic disruptions in carcinoma-bearing rats
Source: Front Microbiol. 2022 Sep 2;13:949272. doi: 10.3389/fmicb.2022.949272 (PMC9479207; doi:10.3389/fmicb.2022.949272)

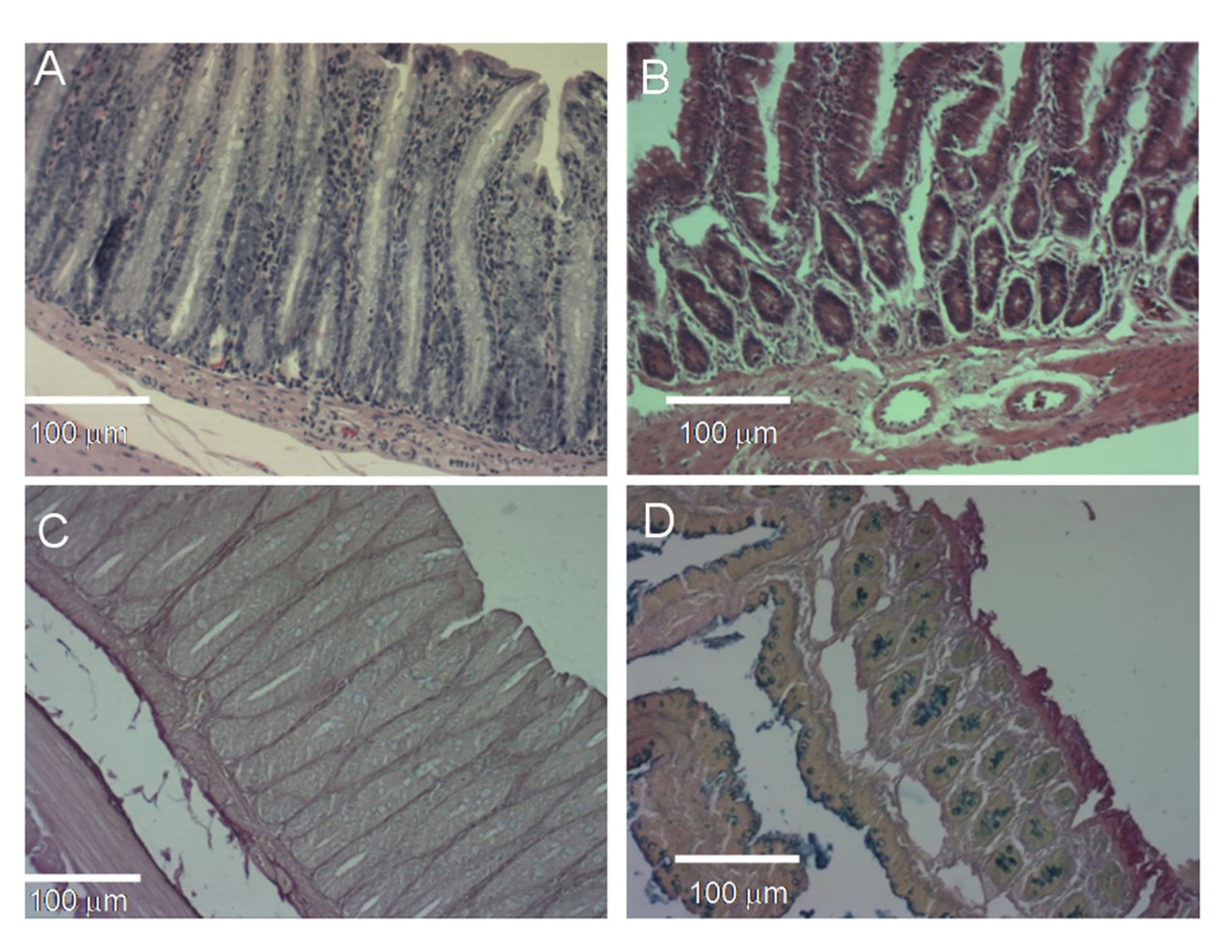

Supplement: Supplementary Figure S1 — Histological section of control (A,C) and with hepatoma (B,D), rat colon stained with hematoxylin-eosin (A,B) or with colloidal iron (C,D). Image analysis was made at 20X. Figure is representative of a total of 6 rat samples. [file Image_1.TIFF]

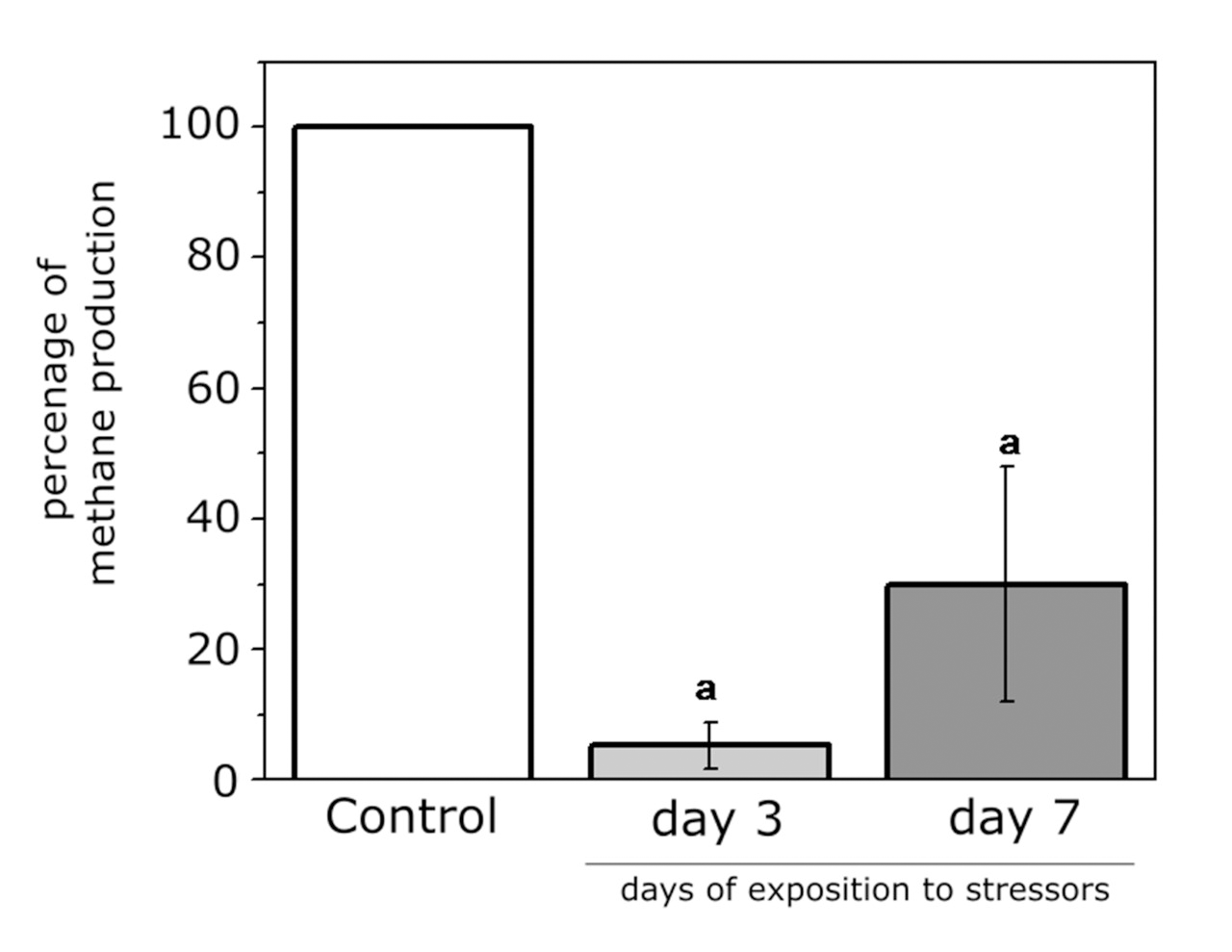

Supplement: Supplementary Figure S2 — Effect of stressors in methane production in cultured microbiota. Control microbiota was cultured in the absence (control, white column) or in the presence of a metal mixture (0.5 mM FeSO4, 0.1 mM CuSO4, 1 mM ZnCl2 and 50 mM KCl) plus 1% (v/v) of O2. Methane production was determined at day 3 (light gray column) and day 7 (gray column) of growth. Values shown are the mean ± SD of 3 independent cultures. a P <0.001 vs. control cultures. [file Image_2.TIFF]

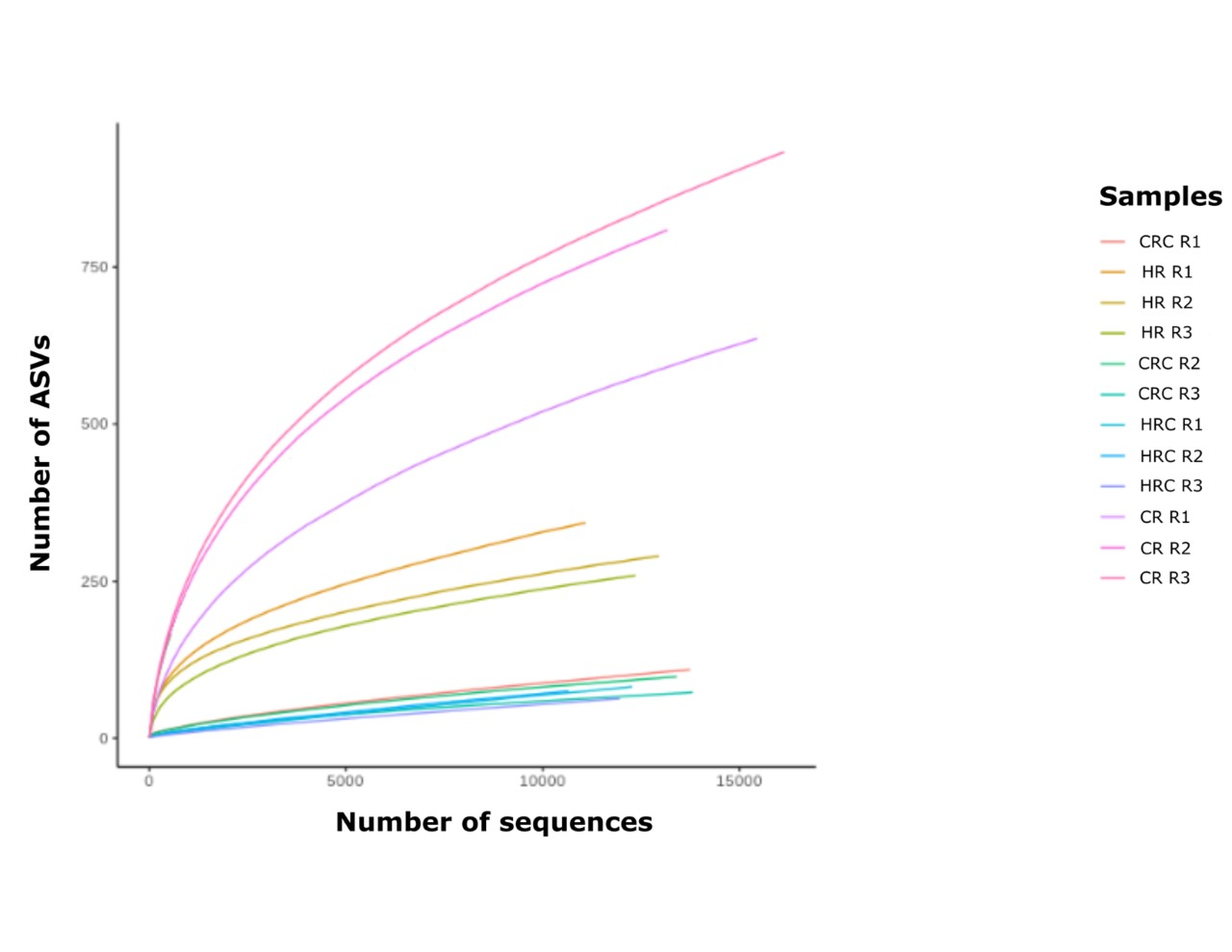

Supplement: Supplementary Figure S3 — Rarefaction plot of amplicon sequences variants (ASVs) from CRC, CR, HRC, and HR. The x axis indicates the number of sequences and y axis indicates the observed microorganisms in ASVs. Each curve represents a different sample replicate. [file Image_3.TIFF]

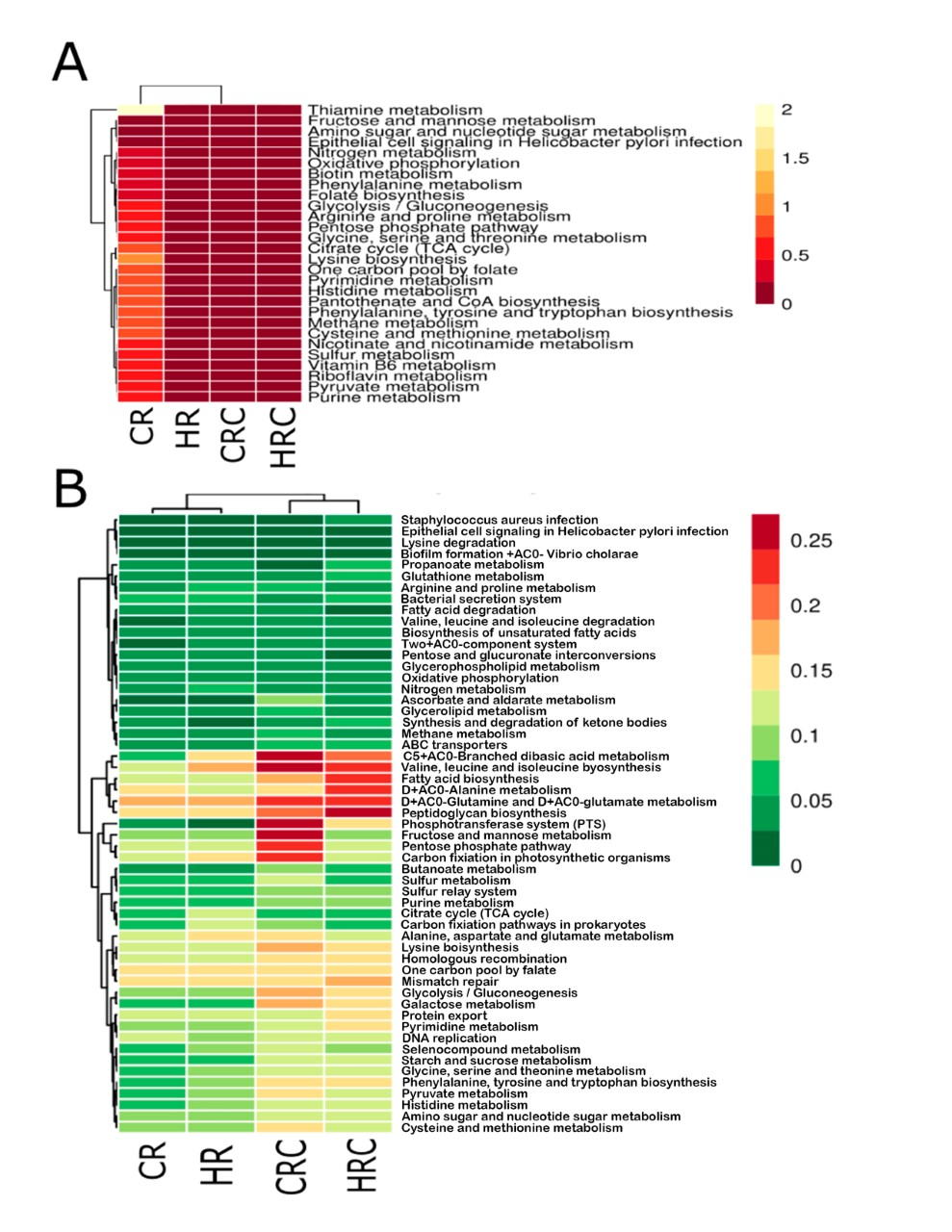

Supplement: Supplementary Figure S4 — Heat map of metabolic function predicted by archaea (A) and bacteria (B) based on community phylogenetic research by reconstructing Unobserved State Analysis (PICRUSt) and the Kyoto Encyclopedia of Genes and Genomes (KEGG). The rows indicate the KEGG level 2 pathway (> 1% abundance) and the columns show the hierarchical groups of the in vivo control model (CR), the AS30D hepatoma in vivo model (HR), the control culture (CRC) and the culture of the microbiota of rats with hepatoma (HRC). [file Image_4.TIFF]

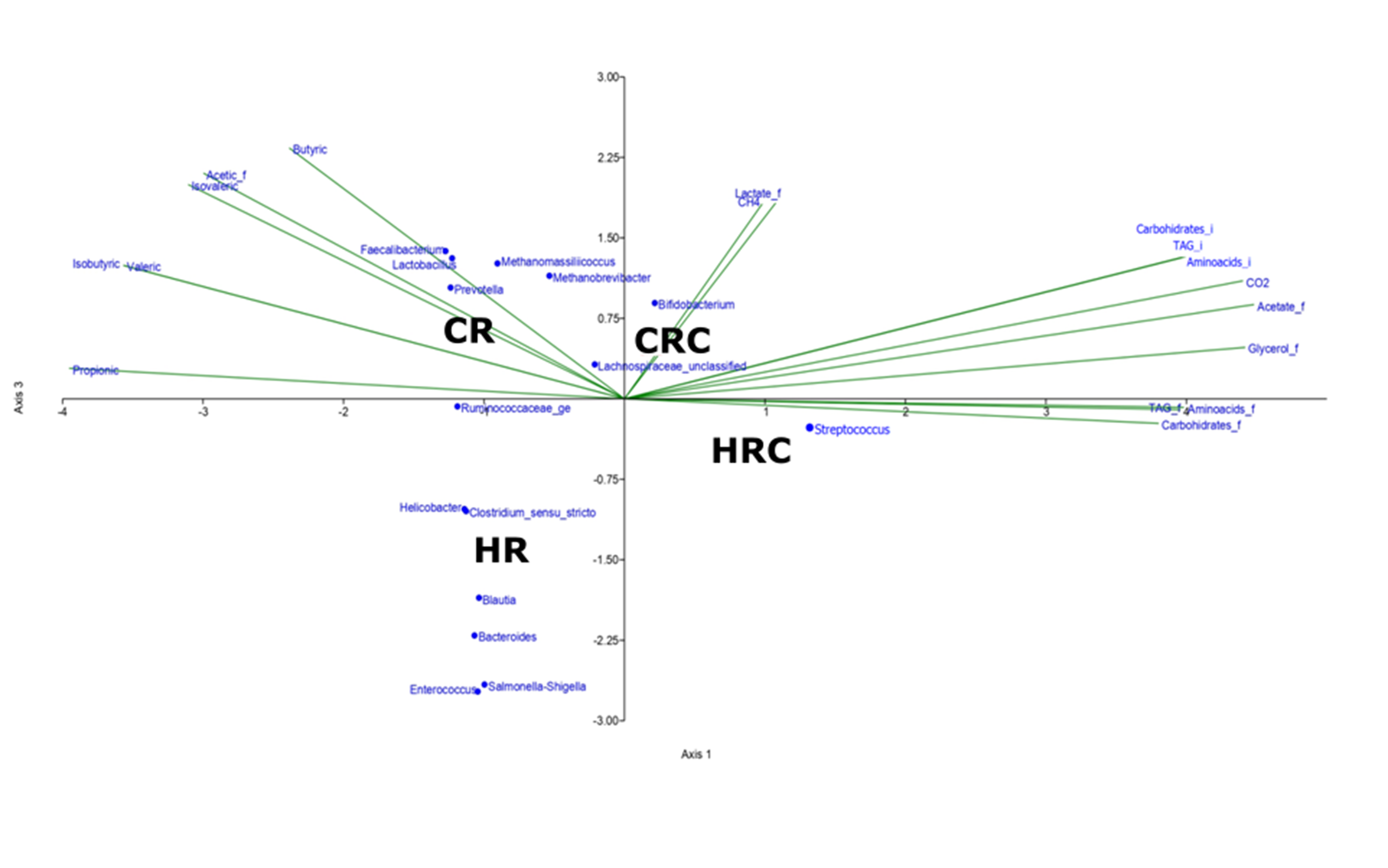

Supplement: Supplementary Figure S5 — Canonical correspondence analysis (CCA) of gut microbial communities and metabolic parameters ASV with relative abundance higher than 0.01% and which is also a marker for microorganisms of intestinal metabolism were applied for CCA, as environmental conditions, the metabolic variables of organic acids, CO2 and methane production, and carbon sources consumption were considered. [file Image_5.TIFF]
