# Supplementary material for: Cultivation of gastrointestinal microbiota in a new growth system revealed dysbiosis and metabolic disruptions in carcinoma-bearing rats
Source: Front Microbiol. 2022 Sep 2;13:949272. doi: 10.3389/fmicb.2022.949272 (PMC9479207; doi:10.3389/fmicb.2022.949272)
Supplement: Supplementary file 6 [file Data_Sheet_1.doc]

S1. Materials

Triacetylglycerol (TAG), sodium pyruvate, glycerol, glucose, lactose, maltose, DL-

lactic acid, enzymatic digest from casein, glutathione, cysteine, sodium sulfide

(nonahydrated), NADH, NAD^+^, NADP^+^ were purchased from Sigma-Aldrich (St

Louis, MO, USA); organic acid standards were from Fluka (Sigma-Aldrich, USA);

dichlorofluorescein diacetate (DCF) was from Thermo-Fisher (Waltham, MA, USA).

Di- and monovalent cations standard solutions for ionomics were from Agilent

(Santa Clara, CA, USA). Sodium acetate, methanol, oxygen peroxide and other

reagents were of analytical grade.

S2. Histopathological analysis

After euthanasia, tissue samples were collected and processed for light

microscopy according to standard techniques. Briefly, a segment of about 2 cm of

intestine (colon) was dissected and washed with saline solution (0.9% w/v NaCl)

for 30 s, fixed in 10% (v/V) formalin solution for 24 h, gradually dehydrated with

ethanol, cleared with xylene, and embedded in paraffin. The intestine was cut into

five-micrometer-thick slices with a microtome (Leica RM212RT; Wetzlar,

Germany); the paraffin slices were stained with hematoxylin-eosin and colloidal-

iron. Histological sections were analyzed at 20× magnification using a model

63300 light microscope (Carl Zeiss; Oberkochen, Germany), equipped with a

Tucsen (9 megapixels) digital camera and analyzed with the TSview 7.3.1

software.

S3.Biofilm synthesis

To determine the ability of the gastrointestinal microbiota (GITm) to form biofilms,

0.2 mL of recently cultured cells were poured into 96 well polystyrene plates and

incubated anaerobically at 37ºC for 2 or 7 days; thereafter biofilm formation was

determined colorimetrically using crystal violet as reported by Zhang et al (2007).

S4. Metals and metabolites determination

For essential metals determination, 0.02-0.05 g DW feces were diluted in 1

mL ultrapure water and digested with 4 mL nitric acid and incubated overnight at

90°C in a dry bath. The samples were later analyzed using an ICP-OES

(inductively coupled plasma - optical emission spectrometer) apparatus (Agilent,

Ca, USA) using Argon as carrier gas.

Fecal occult blood test (FOBT) was determined colorimetrically in 0.25-0.5 g

fresh stools suspended in 0.5 mL deionized water and sequentially adding 0.1 mL

20 % (v/v) acetic acid, 0.1 mL 1 % (w/v) toluidine blue and 0.1 mL H_2_O_2_; a strong

blue color was indicative of a positive reaction. After centrifugation, absorbance of

the supernatants was measured at 600 nm using water as blank.

For short-chain organic acids (SCOA) determination, feces (0.05 - 0.08 g

DW diluted in 1 mL warm ultrapure water) or 1 mL of 8 days-old microbiota cultures

(cells-free) supernatants were withdrawn and mixed with 2.5 mL chloroform *plus*

0.1 mL concentrated HCl and vigorously vortexed for 2 min; afterwards, the

samples were centrifuged at 2,100 *x g* for 5 min and the organic phase was

recovered. Chloroform was evaporated with a N_2_ stream and dried samples were

kept at-20°C until use. Thereafter, samples were thawed and resuspended in 0.1

mL ultrapure water *plus* 5 µL absolute ethanol, after verification that the sample

had a pH value between 2-3, two µL were injected into a gas chromatography

apparatus (GC2010; Shimadzu, Japan) equipped with a DB-1701 capillary column

(Agilent, Santa Clara CA, USA). Acetic, propionic, isobutyric, butyric, valeric and

isovaleric acids were separated, identified and quantified using the respective

standard mix (WSFA-2, Supelco, Munich, Germany) as reported by Romo-Araiza

et al. (2018). Conditions were: oven 225°C, a column ramp from 70°C to 90°C at

3°C/min, the total flow of 36.6 mL/min, FID 230°C and He as carrier gas.

Carbon sources consumption present in the growth media from cultured

microbiota were determined as follows: glucose, lactose and maltose were

quantified as total carbohydrates using the phenol sulfuric acid colorimetric method

(Jasso-Chávez et al., 2015). Methanol, acetate, lactate, glycerol and TAG

consumption was determined by gas chromatography using a FID and a DB1701

capillary column (Agilent, USA) under the following conditions: helium as carrier

gas, oven at 250°C, a column ramp from 65°C to 140°C at 10°C/min and total flow

of 14.7 mL/min and FID 200°C.

Amino acids consumption was determined by using ninhydrin as derivatizing

reagent under acidic conditions as published elsewhere (Friedman, 2004).

Methane produced by archaea cultured in the INC-07 growth media was

determined by gas chromatography and FID using a HP-PLOT U capillary column

(Agilent, Santa Clara, CA, USA) as reported previously by Peña-Ocaña et al.

(2022).

S5. ROS production

Cultured microbiota was harvested under anaerobic conditions at 3000 x *g*

for 15 min; the pellet was resuspended and washed once with 50-75 volumes of

TME buffer (50 mMTris, 20 mM MgCl_2_ and 1 mM EGTA at pH 7.2). Washed cell

suspensions (approximately 1 mL) were kept in 10 mL capacity anaerobic bottles

with Teflon cap at room temperature. ROS production was determined

spectrophotometrically (UV-1800, Shimadzu, Japan) into an anaerobic chamber at

room temperature in a quartz cell with 1.8 mL TME buffer, 0.01 mL stock solution

of carbon sources and 0.5-0.7 mg cellular protein; as probe 0.08 mM DCF

(dichlorofluorescein) was added and the increase in the absorbance was

determined at 504 nm. The rate of ROS production was determined using the

ε_DCF_=90 mM^-1^ cm^-1^ as previously reported (Jasso-Chávez et al., 2015). A control

mix without cellular protein was carried out in parallel and the spurious absorbance

was subtracted from the experimental samples.

S6. Cell extracts preparation and enzyme activities determination

Washed cell suspensions (50-80 mg cell protein) on a pocket with ice, were

disrupted by sonication (3 cycles of 30 s with 2 min of rest); the cell debris was

separated by centrifugation at 7000 *x g* for 10 min. The supernatants were

ultracentrifuged at 100,000 *x g* for 45 min and the supernatant (cytosolic fraction)

and pellet (membrane fraction resuspended in 0.5 mL TME) were mixed with 1 mM

DTT, 1 mM PMSF and 10 % (v/v) glycerol and kept at -70 °C until use.

Enzymatic activities were all determined in 1 mL HKE buffer (25 mM Hepes-

Na, 120 mM KCl, 1 mM EGTA at pH 7.2). Alanine aminotransferase (ALAT),

aspartate aminotransferase (ASAT) and NADPH-producing enzymes (glucose-6P

dehydrogenase, G6PDH; malic enzyme, ME and isocitrate dehydrogenase, IDH)

as well as enzymes involved in the antioxidant response (glutathione reductase

and glutathione peroxidase) were determined spectrophotometrically as previously

reported (Feregrino-Mondragón et al., 2021; Jasso-Chávez et al., 2021). Lactase

and sucrase were determined by coupling the glucose released with commercial

hexokinase (HK) and Glc6PDH (Sigma, St Louis Mo, USA) plus 5 mM ATP and 1

mM NADP^+^ and monitoring the NADPH production at 340 nm in a diode array

detector spectrophotometer (Agilent). Acetate kinase was determined according to

the method published previously by Lira-Silva et al. (2012). NAD^+^-dependent

lactate dehydrogenase (LDH) and membrane-bound iLDH were determined as

reported by Jasso-Chávez et al. (2005). Lipases were determined by using 200 μM

NPA (4-nitrophenyl acetate) and monitoring dinitrophenol release at 400 nm; an

ε_NPA_ of 14 mM^-1^ cm^-1^ was used to calculate the rate. Care was taken that activity

was linear with respect to the protein amount and time; activity in the absence of

their specific substrates was always subtracted.

S7. DNA extraction and library construction

DNA was isolated from 0.1 g fecal samples resuspended in phosphate

buffer solution (PBS; 150 mM NaCl, 10 mM Na_2_HPO_4_, 10 mM NaH_2_PO_4_ at pH 7.4)

or from 10 mL of the enriched fecal microbiota cultures (3^rd^ cultured passage). The

cells were centrifuged at 12,000 *x g* for 10 min and the pellet washed once with 1

mL PBS and centrifuged at 12,000 *× g* for 10 min. DNA was extracted according to

the Quick-DNA ™ kit (Zymo Research, Orange, CA, USA) following the protocol

indicated for cell suspensions and samples digested with proteinase K. Total DNA

|  | amounts obtained were in the intervals of 40-70 and 80-120 ng for feces and |
| --- | --- |
|  | cultured cells, respectively. DNA was lyophilized and sent to the Kyushu Institute of |
|  | Technology, Japan for sequencing. First, DNA integrity was verified by gel |
|  | electrophoresis: 1 µL of sample was mixed with 1 µL of 6X loading dye and 4 µL of |
|  | RNase-free water and subjected to electrophoresis (100 V for 30 min in a 0.8% |
|  | agarose gel). The gel was stained with 0.5 µg x mL^-1^ ethidium bromide for 40 min. |
|  | To amplify the V3-V4 region of the prokaryotic 16S rRNA gene, 12 μL Quick |
|  | Taq (Toyobo; Kamiyoshiba, Satte City, Japan), 9.5 μL sterilized water, 0.75 μL |
|  | (1μM) forward 341primer: (5'- TCGTCGGCAGCGTCAGATGTGTATAAGAGACAG |
|  | CCTACGGGNGWGCAG-3'), 0.75 μL (1 μM) reverse 785 primer: (5'-GTCTCGTGG |
|  | GCTCGGAGATGTGTATAAGAGACAGGACTACHVGGGTATCTAATCC-3') and 2 |
|  | μL DNA as template were used. Amplification conditions were: 94°C 30 s, 55°C 30 |
|  | s, 68°C 1 min, for 30 cycles with a final step of 68°C 5 min. The barcoded DNA |
|  | libraries were sequenced through Illumina's MiSeq 2 × 300 bp platform (San Diego, |
|  | CA, USA). |
|  |  |
|  | **S8. Bioinformatics analyses** |
|  | The bioinformatics analyses were carried out as previously described by Gutiérrez- |
|  | Sarmiento et al. (2020). In detail, the demultiplexed raw sequence data was |
|  | decompressed and quality parameters analyzed with FastQC of paired end reads |
|  | (Andrews et al., 2010). The Usearch (Edgar, 2010) and Vsearch (Rognes et al., |
|  | 2016) tools were used. Forward and reverse reads were merged using |
|  | fastq_merge pairs to create a consensus sequence. Subsequently, the primers |
|  | were cut by 50 bp to the left and 55 bp to the right of each sequence according to |

the respective bp length of the primers. Quality filtering was performed with

fastq_filter based on ± 10% of expected sequence length, fastq_maxlen 444 and

fastq_minlen 330 were applied. Identical sequences were pooled together into one

representative using the derep_full length function followed by the elimination of

chimeras and amplicon errors, for which the unoise3 algorithm was used. A

minimum abundance threshold of 0.05% was established to create the

Amplification Sequence Variant (ASV) table (Parkar et al., 2021). For the count

table, usearch_global with 0.99 threshold similarity was used. The ASV taxonomy

assignment was performed using a non-Bayesian taxonomy classifier and using

the SILVA high quality ribosomal RNA database (silva_16s_v123.fa). In addition,

the reconstruction of unobserved states (PICRUSt) was carried out according to

Langille et al. (2013) , based on the Kyoto Encyclopedia of Genes and Genomes

(KEGG) (Kanehisa et al., 2021) to predict the functional composition of the

metagenome.

Next generation sequencing (NGS) analysis produced a total of 203,589 reads

belonging to theV3-V4 variable regions of the 16S rRNA, an average of 14,496

reads per sample were obtained. Reads of combinations from pair ends were

joined and 175,679 consensus overlapped data were obtained from which 173,055

sequences were retained; their de-replication converged in 45,413 unique

sequences. Metabarcoding readings of 16S rRNA were taxonomically assigned

based on a 97% sequence identity threshold for the identification of gastrointestinal

microbiota genera.

Supplementary References

Andrews, S., Krueger, F., Seconds-Pichon, A., Biggins, F., and Wingett, S. (2010).

Fast QC: A quality control tool for high throughput sequence data. Babraham

Bioinformatics. *Babraham Institute* 1, 1. Available online: .

Edgar, R. C. (2010). Search and clustering orders of magnitude faster than BLAST.

*Bioinformatics* 26, 2460–2461. doi: 10.1093/bioinformatics/btq461.

Feregrino-Mondragón, R. D., Vega-Segura, A., Sánchez-Thomas, R., Silva-Flores,

M., Rodríguez-Zavala, J. S., Marín-Hernández, Á., et al. (2021). The essential role of

mitochondria in the consumption of waste-organic matter and production of metabolites of

biotechnological interest in *Euglena gracilis*. *Algal Res.* 56, 102302. doi:

10.1016/j.algal.2021.102302.

Friedman, M. (2004). Applications of the ninhydrin reaction for analysis of amino

acids, peptides, and proteins to agricultural and biomedical sciences. *J. Agric. Food Chem.*

52, 385-406. doi: 10.1021/jf030490p.

Jasso-Chávez, R., Campos-García, M. L., Vega-Segura, A., Pichardo-Ramos, G.,

Silva-Flores, M., Santiago-Martínez, G. M., et al. (2021). Microaerophilia enhances heavy

metal biosorption and internal binding by polyphosphates in photosynthetic *Euglena*

*gracilis. Algal Res*. 58, 102384. doi: 10.1016/j.algal.2021.102384.

Jasso-Chávez, R., García-Cano, I., Marín-Hernández, Á., Mendoza-Cózatl, D.,

Rendón, J. L., and Moreno-Sánchez, R. (2005). The bacterial-like lactate shuttle

components from heterotrophic *Euglena gracilis*. *Biochim. Biophys. Acta Bioenerg.* 1709,

181-190. doi: 10.1016/j.bbabio.2005.07.007.

Jasso-Chávez, R., Santiago-Martínez, M. G., Lira-Silva, E., Pineda, E., Zepeda-

Rodríguez, A., Belmont-Díaz, J., et al. (2015). Air-adapted *Methanosarcina acetivorans*

shows high methane production and develops resistance against oxygen stress. *PLoS*

*ONE* 10, e0117331. doi: 10.1371/journal.pone.0117331.

Kanehisa, M., Sato, Y., and Kawashima, M. (2021). KEGG mapping tools for

uncovering hidden features in biological data. *Protein Sci.* 31, 47-53. doi:

10.1002/pro.4172.

Langille, M. G. I., Zaneveld, J., Caporaso, J. G., McDonald, D., Knights, D., Reyes,

J. A., et al. (2013). Predictive functional profiling of microbial communities using 16S rRNA

marker gene sequences. *Nat. Biotechnol.* 31, 814-821. doi: 10.1038/nbt.2676.

Lira-Silva, E., Santiago-Martínez, M. G., Hernández-Juárez, V., García-Contreras,

R., Moreno-Sánchez, R., and Jasso-Chávez, R. (2012). Activation of methanogenesis by

cadmium in the marine archaeon *Methanosarcina acetivorans*. *PLoS ONE* 7, e48779. doi:

10.1371/journal.pone.0048779.

Parkar, S.G., Frost, J., Rosendale, D., Stoklosinski, H. M., Jobsis, C. M. H., Hedderley, D. I., and Gopal, P. (2021). The sugar composition of the fibre in selected plant foods modulates weaning infants’ gut microbiome composition and fermentation metabolites in vitro. Sci Rep 11, 9292. <https://doi.org/10.1038/s41598-021-88445-8>.

Pinho, S. C. M., Faria, M. A., Melo, A., Pinto, E., Almeida, A., Alves, R., et al.

(2021). Effect of skimmed milk on intestinal tract: Prevention of increased reactive oxygen

species and nitric oxide formation. *Int. Dairy J.* 118, 105046. doi:

10.1016/j.idairyj.2021.105046.

Rognes, T., Flouri, T., Nichols, B., Quince, C., and Mahé, F. (2016).VSEARCH: A

versatile open source tool for metagenomics. *Peer J.* 4, e2584. doi: 10.7717/peerj.2584.

Zhang, X. S., García-Contreras, R., and Wood, T. K. (2007). YcfR (BhsA)

influences *Escherichia coli* biofilm formation through stress response and surface

hydrophobicity. *J. Bacteriol*. 189, 3051–3062. doi: 10.1128/JB.01832-06.
